# Review of antipsychotic prescribing at HMP/YOI Low Newton

**DOI:** 10.1192/bjb.2020.80

**Published:** 2022-02

**Authors:** Lois Carey, Stephen Barlow

**Affiliations:** 1 Roseberry Park Hospital, Middlesbrough, UK

**Keywords:** Antipsychotics, prescribing, prison, polypharmacy, off label

## Abstract

**Aims and Method:**

The purpose of this review was to establish whether the prescription of antipsychotic medication in HMP Low Newton was safe, rational and consistent with current best practice. A search of the electronic healthcare records was performed on 14 March 2018 to identify all the women in the prison who were prescribed antipsychotic medication, and then data were collected from the records.

**Results:**

A total of 46 out of 336 prisoners (13.7%) had been prescribed antipsychotic medications; 29 of the 46 patients (84.8%) were also prescribed other psychotropic medications at the same time. Quetiapine was the most frequently prescribed antipsychotic and was also the most likely to be prescribed for off-label indications. Less than one-third of all antipsychotic prescriptions were for psychotic disorders.

**Clinical implications:**

The rationale for prescribing all antipsychotic medication, especially for off-label indications, should be clearly documented and reviewed regularly within the prison by the mental health team and psychiatrist.

## Introduction

HMP Low Newton is one of 13 women's prisons in England, and houses approximately 350 inmates aged 18 years and above. It manages a mixed population of remand and short-term sentenced prisoners and those serving longer sentences, including a significant number serving life or indeterminate sentences. The turnover is high (approximately 70 receptions per week) and psychiatric morbidity is common.

Women in prison are five times more likely to have a mental health problem than women in the general population, with 78% exhibiting some kind of psychological disturbance on reception according to the 12-item General Health Questionnaire.^1^ A study of remanded prisoners found 11% to be acutely psychotic on reception.^2^

At any one time, the mental health team in HMP Low Newton provides support to about a third of the population, about a quarter of whom have complex mental health needs.^3^ The team conducts triage assessments for approximately 20 new patients each week. There are three sessions of clinical input from a psychiatrist, which, given the constraints on access to prisoners, amounts to approximately 6–8 h direct clinical contact per week. There are no other prescribers within the team. Primary care services are provided by an independent organisation, G4S Healthcare.

Antipsychotics are potent drugs, primarily licensed for the management of schizophrenia, mania and other severe psychotic disorders. They have the potential to cause a wide range of acute and long-term side-effects, some of which can be serious, including parkinsonism, neuroleptic malignant syndrome, obesity, diabetes, cardiotoxicity, sudden death, hormonal changes, electrolyte imbalances, convulsions and blood dyscrasias.

Although they can also be prescribed in both psychiatric and general practice for the relief of stress, anxiety and psychological distress, those licensed for this purpose, which include chlorpromazine, olanzapine and haloperidol, should only be used in the short term. There is no evidence that antipsychotics have any demonstrable long-term benefit in the management of personality disorders.^4^ Despite this, off-label prescribing is increasingly common and has been identified as a cause for concern.^5^

There is a growing body of literature highlighting the misuse potential of some antipsychotic medications, particularly quetiapine.^6,7^ There is also increasing awareness of the risks of harm from misuse and diversion of prescribed medicines in prison. These extend beyond the effects of the drugs themselves to include bullying, threats and coercion, and debt.^8,9^ Deaths from the misuse of prescribed drugs, including antipsychotics, are rising. The numbers of deaths caused by antipsychotics in 2017 saw an increase of just over 5% from 2016, which equates to a rate of 2.1 per 1 million population.^10^

## Aims/objectives

The purpose of this review was to ascertain whether the prescription of antipsychotic medication in HMP Low Newton was safe, rational and consistent with current best practice. We considered best practice guidance from the National Institute for Health and Care Excellence and adopted the following principles.

- Antipsychotic medication should only be prescribed if there is a clearly documented, evidence-based rationale, consistent with the drug's licence and/or best practice guidelines.
- Patients receiving antipsychotic drugs should be regularly reviewed by specialist mental health services.
- No patient should be prescribed more than one antipsychotic or equivalent doses above *British National Formulary* (BNF) limits.

## Method

This review was registered with and approved by Tees, Esk and Wear Valleys (TEWV) NHS Foundation Trust clinical audit department.

All medical records within the prison are held on an electronic system, and all prescribing is done electronically. A search of the electronic healthcare records was performed on 14 March 2018 to identify all the women in the prison who were being prescribed antipsychotic medication on that day. Their healthcare records were then reviewed by the authors to identify:

- patient's age
- date of reception into HMP Low Newton
- antipsychotic prescribed and dose
- prescriber's professional status
- date of prescription
- diagnosis or indication for prescription (where recorded)
- other psychotropic medication
- past or future contact with mental health services.

Within TEWV NHS Foundation Trust, ‘off label’ is defined as the use of a medicine that has a marketing authorisation for an indication (condition), at a dose, via a route or for a patient category (e.g. age) that is not listed in the Summary of Product Characteristics for that medicine. Therefore, any antipsychotic prescription that met this criterion was defined as ‘off label’.

## Results

The prison roll for that day was 336. Forty-six women (13.7% of the total population) were identified as having a current prescription for antipsychotic medication.

### Age distribution

**Fig. 1:**
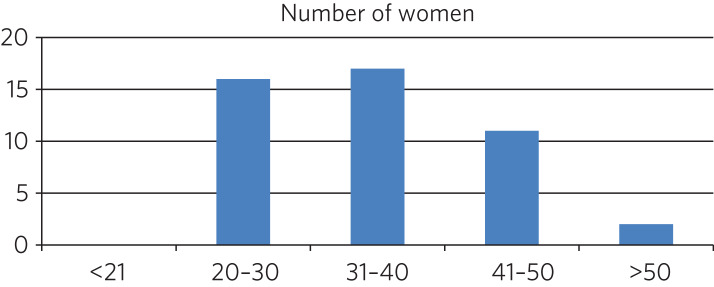
Age in years of the women prescribed antipsychotic medication

The ages of the women taking antipsychotic medication ranged from 22 to 55 years, with a mean and median of 35 years (Fig. 1 and Table 1) This was consistent with the age profile of the population as a whole within the prison.^3^

**Table 1:** Age distribution

Age, years	Patients, *N*
<21	0
21–30	16
31–40	17
41–50	11
>50	2

### Length of time in prison

The number of days each patient had been in HMP Low Newton on the date of the audit ranged from 4 to 2430, with an average of 363 days. The distribution suggested distinct groups, with the majority having spent less than 180 days in the prison and a second significant group having been in for more than a year, reflecting the fact that the prison houses both a transient population of remand and short-sentenced prisoners and a more stable population serving longer-term sentences (Table 2).

**Table 2:** Length of time in prison in days

Time in prison, days	No. of prisoners
<91	11
91–180	11
181–270	4
271–360	6
>361	14
Range	4–2430 days
Average	363 days

### Reception

The majority of patients (26, 56.5%) had been admitted directly from the community; 19 (41.3%) had come from other prisons and one (2.2%) had been discharged from a psychiatric hospital.

### Antipsychotics prescribed

The antipsychotics prescribed are shown in Table 3. By far the most popular antipsychotics were quetiapine (20 patients) and olanzapine (16 patients). Other antipsychotics prescribed were risperidone (three patients), flupenthixol (two patients), aripiprazole (two patients), sulpride (one patient), chlorpromazine (one patient) and zuclopenthixol (one patient) (Table 3).

**Table 3:** Antipsychotics prescribed

Antipsychotic	No. of patients
Quetiapine (inc. modified release)	20 (43.5%)
Olanzapine	16 (34.8%)
Risperidone	3 (6.50%)
Sulpride	1 (2.20%)
Flupenthixol	2 (4.30%)
Aripiprazole	2 (4.30%)
Chlorpromazine	1 (2.20%)
Zuclopenthixol	1 (2.20%)

No patients were prescribed doses above BNF limits. One patient was prescribed two antipsychotics, zuclopenthixol and aripiprazole, after it was recommended that aripiprazole be started for hyperprolactinaemia secondary to zuclopenthixol.

### Prescriber

Thirty (65.20%) of the prescriptions for antipsychotics were started by a psychiatrist. These included 12 (26.10%) started in HMP Low Newton during the current period of imprisonment; three (6.50%) had been started in another prison but during the current period of imprisonment, and 15 (32.5%) had been started in the community or during a previous period of imprisonment. Sixteen (34.80%) prescriptions were prescribed by a general practitioner (GP) or primary care services.

### Polypharmacy

One patient was prescribed aripiprazole for hyperprolactinaemia in addition to zuclopenthixol. Only seven patients (15.2%) were not prescribed any additional psychotropic medications.

The amounts and types of other psychotropic medications prescribed are summarised below (Tables 4 and 5).

**Table 4:** Number of additional psychotropics prescribed

Additional psychotropics	No. of patients
0	7
1	27
2	10
3	2
4	0
5	0

**Table 5:** Type of additional psychotropics prescribed

Type of drug	No. of patients	No. of scripts	Specific agent	Scripts for agent
Antidepressant	29	32	Mirtazapine	13
			Sertraline	7
			Trazadone	4
			Fluoxetine	3
			Venlafaxine	2
			Amitriptyline	1
			Duloxetine	1
			Paroxetine	1
				
Opiates	14	14	Methadone	13
			Subutex	1
				
Benzodiazepine	2	2	Clonazepam	2
				
Mood stabilisers/anticonvulsants	3	3	Depakote	1
			Lamotrigine	2
				
Stimulants	2	2	Atomoxetine	1
			Dexamphetamine	1

One patient was prescribed a total of five psychotropic medications, comprising zuclopenthixol depot injection, aripiprazole, atomoxetine, sertraline and clonazepam. This patient had been returned to prison from a medium secure unit and had a diagnosis of emotionally unstable personality disorder (EUPD) and attention-deficit hyperactivity disorder.

One patient was prescribed three psychotropic medications (duloxetine, lamotrigine and buprenorphine) in addition to quetiapine. Medication had been started in HMP New Hall, and the diagnosis recorded was depression and EUPD.

### Indication and diagnosis

The BNF licensed indications for each of the prescribed antipsychotics prescribed are summarised in Table 6.

**Table 6:** BNF licence indication for each antipsychotic prescribed

BNF licence indication	Aripiprazole	Chlorpromazine	Flupenthixol	Olanzapine	Quetiapine (inc modified release)	Risperidone	Sulpride	Zuclopenthixol
Schizophrenia/psychosis	x	x	x	x	x	x	x	x
Mania	x	x		x	x	x		
Short-term management of agitation/excitement/anxiety		x		x				
Depression			x		x			
Bipolar prophylaxis					x			
Nausea/vomiting/tics/hiccup								
Short-term use for aggression in patients with Alzheimer's						x		

Patient notes were reviewed to identify recorded diagnoses or indications for each patient who had been prescribed an antipsychotic. The reasons recorded in the notes are summarised in Tables 7 and 8.

**Table 7:** Documented indication for each antipsychotic medication in the notes

	Quetiapine	Olanzapine	Risperidone	Sulpride	Flupenthixol	Aripiprazole	Chlorpromazine	Zuclopenthixol	Total
EUPD	10	4	2	1	1		1	1	20
EUPD + PTSD	2	1							3
EUPD + depression	1	1							2
EUPD + psychosis		2							2
**Psychosis/schizophrenia**	2	3			1	1			7
**Drug-induced psychosis**		3							3
**Bipolar**	2	1							3
**Mood stabilisation**	2					1			3
**Depression**									0
**Psychotic depression**			1						1
**Anxiety/ paranoia**	1	1							2
**Number**	20	16	3	1	2	2	1	1	46

**Table 8:** Licenced versus off-label indications documented in the notes for each antipsychotic prescription

Antipsychotic (*N*)	Licensed indication (*N*)	Off-label indication (*N*)
Quetiapine (20)	Depression (1)	Anxiety/paranoia(1)
	Psychosis/schizophrenia (2)	EUPD (10)
	Bipolar (2)	EUPD + PTSD(2)
		Mood stabilisation (2)
Olanzapine (16)	Psychosis/schizophrenia (5)	EUPD (4)
	Drug-induced psychosis (3)	EUPD + PTSD (1)
	Anxiety/paranoia (1)	Bipolar (1)
		Depression (1)
Risperidone (3)		EUPD (2)
		Psychotic depression (1)
Sulpride (2)		EUPD (1)
		Anxiety/paranoia (1)
Flupenthixol (2)	Psychosis/schizophrenia (1)	EUPD (1)
Aripiprazole (1)	Psychosis (1) (hyperprolactinaemia)	
Chlorpromazine (1)		EUPD (1)
Zuclopenthixol depot (1)		EUPD (1)

Less than a third (13/46 = 28.3%) of the prescriptions for antipsychotics were for psychosis (including affective psychoses), and three were for bipolar affective disorder. The remainder were for non-psychotic conditions.

Sixteen of the 46 prescriptions were within the licensed indications. Of the 30 (65.2%) that were prescribed for off-label indications, half (15/30 = 50%) were quetiapine (Table 9). Fisher's exact test was used to determine the significance of this and gave a *P*-value of 0.3496, which is below the typical cut-off for statistical significance (*P* < 0.05).

**Table 9:** Number of off label prescriptions for quetiapine in comparison to other antipsychotics

	Licensed indication	Off-label indication
Quetiapine	5	15
Other drugs	11	15

Two-thirds of the prescriptions where non-licensed indications were recorded in relation to quetiapine were for EUPD (10/15 = 66.6%), plus additional two for EUPD and post-traumatic stress disorder (12/15 = 80%).

### Contact with mental health team

**Fig. 2:**
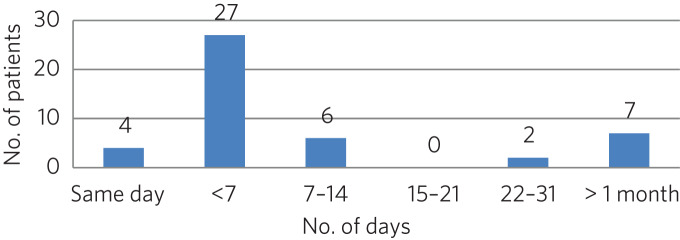
Length of time between arrival in HMP Low Newton and review by the mental health team

**Fig. 3:**
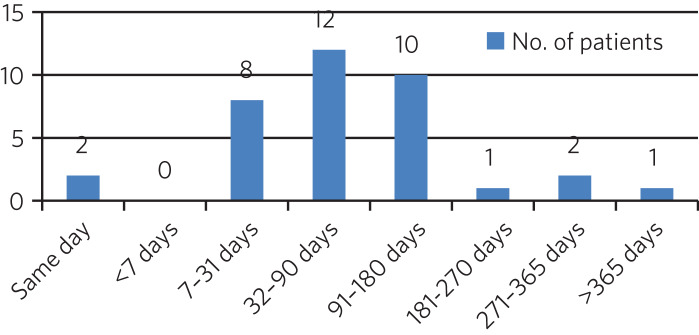
Length of time between review with the mental health team and review with a psychiatrist

The average length of time between arrival in HMP Low Newton and review by the mental health team was 30.2 days (range 0–310 days). This is summarised further in Fig. 2. The average number of days between arrival and review for the seven patients who were seen more than 31 days after coming to HMP Low Newton was 166 days, which suggests that these patients tended to have longer sentences and did not present with symptoms until later in their sentences. Of the 46 patients, 36 had been reviewed by a psychiatrist, five were on the waiting list to be seen, one had declined input as they felt mentally stable and four had no follow-up planned. The average time between review by the mental health team and review by a psychiatrist was 87.97 days. This is broken down further in Fig. 3.

Of the five patients on the waiting list for the psychiatrist, four had been seen by the mental health team for the first time in the 10 days prior to the sample collection date (14 March 2018). One patient had been on the waiting list since January 2018 and had been waiting 8 weeks so far for an appointment. Of the four patients that had not been seen by the psychiatrist and were also not on the waiting list to be seen, 50% (2/4) continued to be followed up by the mental health team. All four of these patients were prescribed quetiapine which had been commenced prior to coming to prison. The characteristics of these four patients are outlined in more detail in Table 10.

**Table 10:** The characteristics of the 4 patients prescribed antipsychotic medications but not under psychiatrist review

	Patient 1	Patient 2	Patient 3	Patient 4
Age (years)	36	35	49	35
Time in HMP Low Newton (days)	120	141	16	313
**Received from**	HMP New Hall	Community	Community	Community
**Antipsychotic**	Quetiapine	Quetiapine	Quetiapine	Quetiapine
**Other psychotropics**	Mirtazapine	Methadone	nil	Mirtazapine
**Antipsychotics commenced**	Prior to reception	Prior to reception	Prior to reception	Prior to reception
**Diagnosis**	EUPD	Psychotic episode	EUPD	Anxiety, depression, antisocial personality disorder
**Follow-up by mental health team**	No	No	Yes	Yes

## Summary of findings

- On the day of the survey, 46/336 prisoners (13.7%) were prescribed antipsychotic medications.
- No patients were prescribed high-dose antipsychotics.
- Only one patient was prescribed more than one antipsychotic, and the rationale for this was clearly stated.
- Twenty-nine of the 46 patients (84.8%) were prescribed other psychotropic medications, most commonly an antidepressant and/or an opiate.
- Two patients (4.34%) were prescribed four or more psychotropic medications. Both of these had diagnoses of EUPD.
- Approximately a third of prescriptions were initiated by primary care/GP.
- Of the 65.2% of prescriptions initiated by psychiatrists, approximately half were started during the current period of imprisonment.
- Quetiapine was the most frequently prescribed antipsychotic and was also the most likely to be prescribed for off-label indications.
- Less than a third of all antipsychotic prescriptions were prescribed to treat psychotic disorders.
- The most common non-licensed indication for antipsychotics being prescribed was EUPD, which accounted for approximately half of all the prescriptions.
- The average length of time between arrival in HMP Low Newton and review by the mental health team was 30 days, with the majority being seen in less than 7 days.
- Forty-one of the 46 patients (89.1%) had prior or planned appointments with the psychiatrist.

## Discussion

This review identified a number of positive findings: no patient was prescribed high-dose antipsychotic therapy; only one patient was prescribed combination antipsychotic therapy, and in this case the second agent was commenced for a side-effect of the first and had been initiated by a psychiatrist who remained involved in the patient's care. The majority of patients were reviewed by the mental health team with 7 days of reception and had prior or planed appointments with the psychiatrist to review their medication.

The review highlighted a high rate of off-label prescriptions for antipsychotic medication within the prison. The study confirmed that this was particularly the case with quetiapine, which, given the high rates of misuse of this drug within the prison, is of concern. Although there was no clear reason for this, one can speculate that, owing to the high number of female prisoners reporting difficulties with ‘mood’ and ‘voices’, quetiapine is chosen because of its reported benefits in relation to ‘mood stabilisation’, as well as its antipsychotic effects. There is also, however, a likelihood that this medication is often requested by the prisoners for its ‘tradeable’ status and potential for misuse.^11^

There is a high rate of polypharmacy in relation to psychotropic medication, which can increase the risk of side-effects and physical health complications. All but two of the patients were prescribed no more than three psychotropic agents. Again, this is likely to be due in part to a high rate of comorbid substance misuse within the prison population. Furthermore, there is a tendency for patients to prefer medication over alternative treatments such as psychological intervention.

Psychological therapies are identified as the primary treatment for patients with personality disorder.^4^ At HMP Low Newton, a variety of psychological services are available. These include the 12-bedded Primrose Unit, for women with severe personality disorder, which forms part of the Offender Personality Disorder Pathway, and a Psychologically Informed Planned Environment wing. The prison forensic psychology services offer a range of assessments and treatments, and the scope of this work is driven by consultancy with offender management units and offender managers within the community. Finally, there is the prison mental health team, who work in line with trauma-informed care principles and can offer a range of individual and group therapies. However, there is often a waiting list for such services, and potentially suitable prisoners often do not remain within the prison long enough to start and complete identified treatments before release or transfer.

Although a large proportion of the prescriptions were issued by a psychiatrist, it is noted that approximately half were commenced in either in the community or during a different prison sentence. Therefore, it is possible that some patients are reissued prescriptions without a thorough review of need, current mental state and other prescribed treatments. Also, the records indicate that often little consideration is given to whether the patient has adhered to medication in the community and so, often, this is simply re-prescribed if it is on the GP summary. The guidelines in relation to use of antipsychotic medications for minor symptoms, such as anxiety, stress or agitation, are clear that it should be a short-term measure only. However, such prescriptions are often continued for prolonged periods without a critical review. This tendency may be exacerbated in the prison population by transfers to other establishments, early release, failures to engage with the mental health team, and a relative lack of attention paid to the initial timing and indications for prescribing.

## Recommendations

- The patient's diagnosis and/or the indication for each prescription should be clearly recorded at the point of prescription in the running case record and in the medication section of the electronic notes system.
- In order to reduce the rate of off-label prescribing, all patients arriving at the prison who are prescribed antipsychotic medication on or shortly after reception should be brought to the attention of the psychiatrist and the mental health team manager.
- All of these patients should be allocated to a secondary care worker and given an appointment to see the team psychiatrist.
- The secondary care nurse should obtain all relevant past records.
- The psychiatrist should conduct an initial case review note and advise on either withdrawing or continuing antipsychotic medication prior to the review.
- Particular attention should be given to patients who have a primary diagnosis of EUPD, particularly those for whom antipsychotics have been prescribed for the relief of minor symptoms, such as stress, anxiety and insomnia. The presumption should be that medications for these reasons should only be prescribed in the short term, and this should be clearly explained to the patient.
- Care should also be taken with patients who have a history of substance misuse, or of secreting and hoarding medication, as this often indicates involvement in trading.
- If patients have not adhered to antipsychotic medication in the community, then it should not be automatically re-prescribed on reception without review by the mental health team or psychiatrist.
- Prescriptions for quetiapine should be kept under regular review and withdrawn unless there are clear reasons for continuing to prescribe.
- This review should be repeated after a year to assess the effects of these measures on off-label prescribing.

## About the authors

**Lois Carey** is a consultant forensic psychiatrist at Roseberry Park Hospital, Middlesbrough, UK. **Stephen Barlow** is a consultant forensic psychiatrist at Roseberry Park Hospital, Middlesbrough, UK.
